# CT-based thermometry with virtual monoenergetic images by dual-energy of fat, muscle and bone using FBP, iterative and deep learning–based reconstruction

**DOI:** 10.1007/s00330-021-08206-z

**Published:** 2021-07-29

**Authors:** Andreas Heinrich, Sebastian Schenkl, David Buckreus, Felix V. Güttler, Ulf K-M. Teichgräber

**Affiliations:** 1 Department of Radiology, Jena University Hospital – Friedrich Schiller University, Am Klinikum 1, 07747 Jena, Germany; 2 Institute of Forensic Medicine, Jena University Hospital – Friedrich Schiller University, Am Klinikum 1, 07747 Jena, Germany

**Keywords:** Computed tomography, Thermometry, Dual-energy scanned projection, Deep learning

## Abstract

**Objectives:**

The aim of this study was to evaluate the sensitivity of CT-based thermometry for clinical applications regarding a three-component tissue phantom of fat, muscle and bone. Virtual monoenergetic images (VMI) by dual-energy measurements and conventional polychromatic 120-kVp images with modern reconstruction algorithms adaptive statistical iterative reconstruction-Volume (ASIR-V) and deep learning image reconstruction (DLIR) were compared.

**Methods:**

A temperature-regulating water circuit system was developed for the systematic evaluation of the correlation between temperature and Hounsfield units (HU). The measurements were performed on a Revolution CT with gemstone spectral imaging technology (GSI). Complementary measurements were performed without GSI (voltage 120 kVp, current 130–545 mA). The measured object was a tissue equivalent phantom in a temperature range of 18 to 50°C. The evaluation was carried out for VMI at 40 to 140 keV and polychromatic 120-kVp images.

**Results:**

The regression analysis showed a significant inverse linear dependency between temperature and average HU regardless of ASIR-V and DLIR. VMI show a higher temperature sensitivity compared to polychromatic images. The temperature sensitivities were 1.25 HU/°C (120 kVp) and 1.35 HU/°C (VMI at 140 keV) for fat, 0.38 HU/°C (120 kVp) and 0.47 HU/°C (VMI at 40 keV) for muscle and 1.15 HU/°C (120 kVp) and 3.58 HU/°C (VMI at 50 keV) for bone.

**Conclusions:**

Dual-energy with VMI enables a higher temperature sensitivity for fat, muscle and bone. The reconstruction with ASIR-V and DLIR has no significant influence on CT-based thermometry, which opens up the potential of drastic dose reductions.

**Key Points:**

• *Virtual monoenergetic images (VMI) enable a higher temperature sensitivity for fat (8%), muscle (24%) and bone (211%) compared to conventional polychromatic 120-kVp images.*

*• With VMI, there are parameters, e.g. monoenergy and reconstruction kernel, to modulate the temperature sensitivity. In contrast, there are no parameters to influence the temperature sensitivity for conventional polychromatic 120-kVp images.*

• *The application of adaptive statistical iterative reconstruction-Volume (ASIR-V) and deep learning–based image reconstruction (DLIR) has no effect on CT-based thermometry, opening up the potential of drastic dose reductions in clinical applications.*

## Introduction

**Fig. 1:**
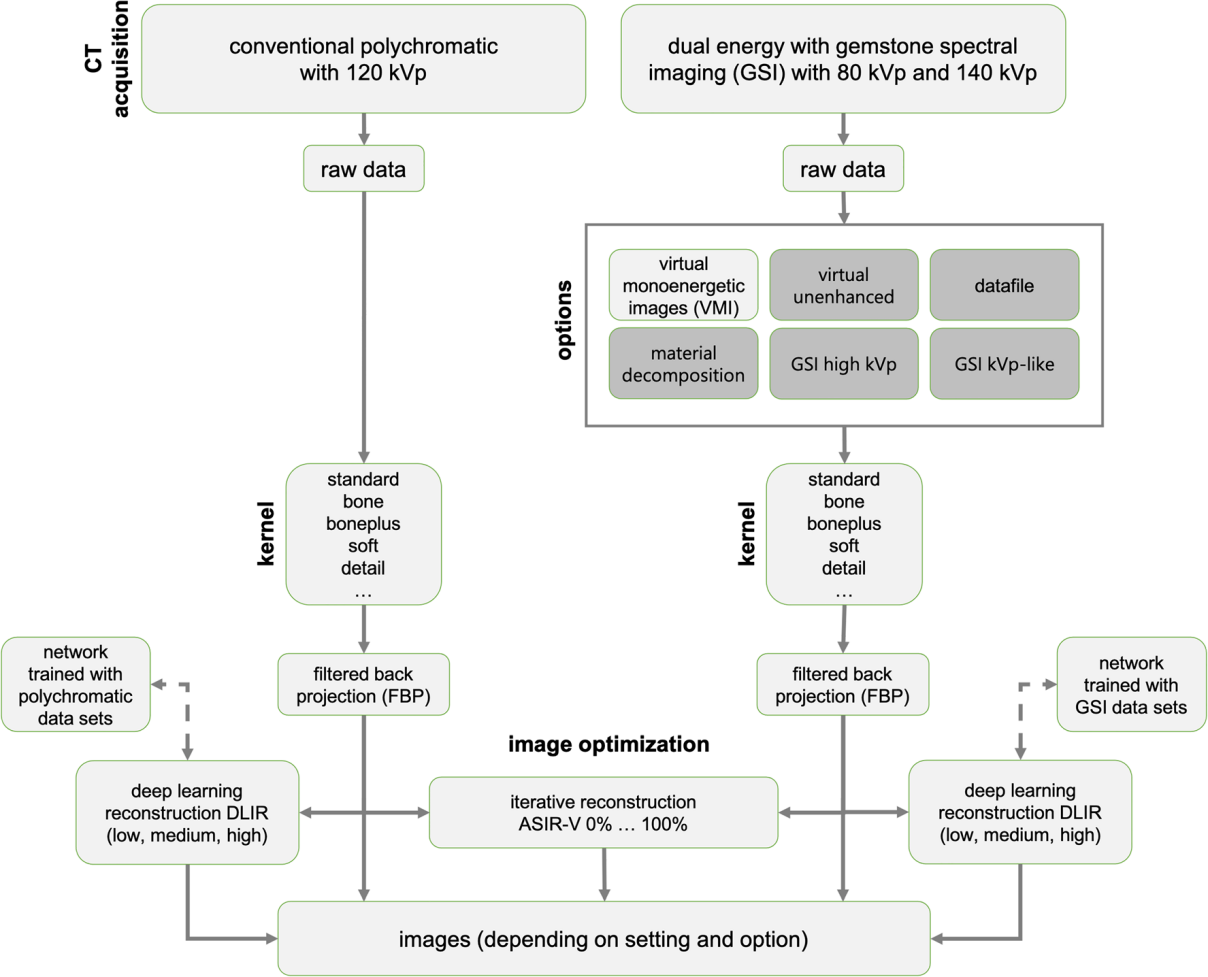
Scheme of data acquisition and image reconstruction in the CT.

Radiodensity depends on the temperature of the tissue, since the density decreases if the temperature of the tissue increases. The changes in tissue temperature can therefore be measured indirectly via the computed tomography (CT) numbers or Hounsfield units (HU), also called CT-based thermometry. However, the sensitivity (change of HU per °C) depends on the volumetric thermal expansion coefficient of the specific material [1]. The temperature sensitivity was reported to range between −0.40 and −0.29 HU/°C for water [1], between −0.52 and −0.60 HU/°C for porcine liver [2, 3], and between −0.45 and −0.43 HU/°C for muscle [4, 5]. Possible applications of CT-based thermometry would be the monitoring of tissue temperatures during high-frequency treatments or microwave ablation of tumors in the liver and kidneys [2, 3, 6–9]. Despite these clear relationships, the method is rarely used in clinical practice so far [10], because the repeatability of quantitative CT numerical measurements has not been guaranteed and a higher required ionizing radiation, e.g. for thermometry due to more frequent scans [11]. Further research is required to systematically analyze the influencing factors on CT-based thermometry [12, 13], so as demonstrate if better levels of thermal-spatial resolution could be achieved, along with lower patient radiation doses [1]. With recent technical advances in CT (Fig. 1), such as dual-energy and iterative reconstruction, e.g. adaptive statistical iterative reconstruction-Volume (ASIR-V) [14, 15], or deep learning image reconstruction (DLIR) [16], the question arises whether these advances have improved the quality of CT-based thermometry. DLIR represents the latest stage of development in CT image reconstruction and promises significant improvements in image quality and radiation dose reduction compared to ASIR-V and filtered back projection (FBP) [16–18]. A dual-energy CT with a gemstone spectral imaging (GSI) detector measures the raw data of an object during a continuous energy change between low (80 kVp) and high (140 kVp) voltage [19]. This generates two data records with different attenuation values based on the corresponding energy levels, which allow the reconstruction of material decomposition images and virtual monoenergetic images (VMI). VMI demonstrated significantly better signal-to-noise ratio (SNR), contrast-to-noise ratio (CNR) and subjective score compared to conventional 120-kVp polychromatic images [20].

The objective of this study was to evaluate CT-based thermometry for fat, muscle and bone using state-of-the-art imaging technology. This includes the dose reduction potential of modern reconstruction algorithms with ASIR-V and DLIR, and the use of VMI at 40–140 keV versus conventional polychromatic 120-kVp images.

## Materials and methods

The measured object was a tissue-equivalent phantom [21, 22], composed of beeswax (density 0.95 g/cm^3^, fat-equivalent), salt-water solution (density 1.04 g/cm^3^, muscle-equivalent) and a bovine femoral bone (density 1.36 g/cm^3^). The phantom (length 19.40 cm, width 17.20–17.40 cm and height 15.20 cm) was placed into a separate phantom container (length 39.00 cm, width 32.00 cm and height 30.50 cm) and fastened.

### Temperature regulation

**Fig. 2:**
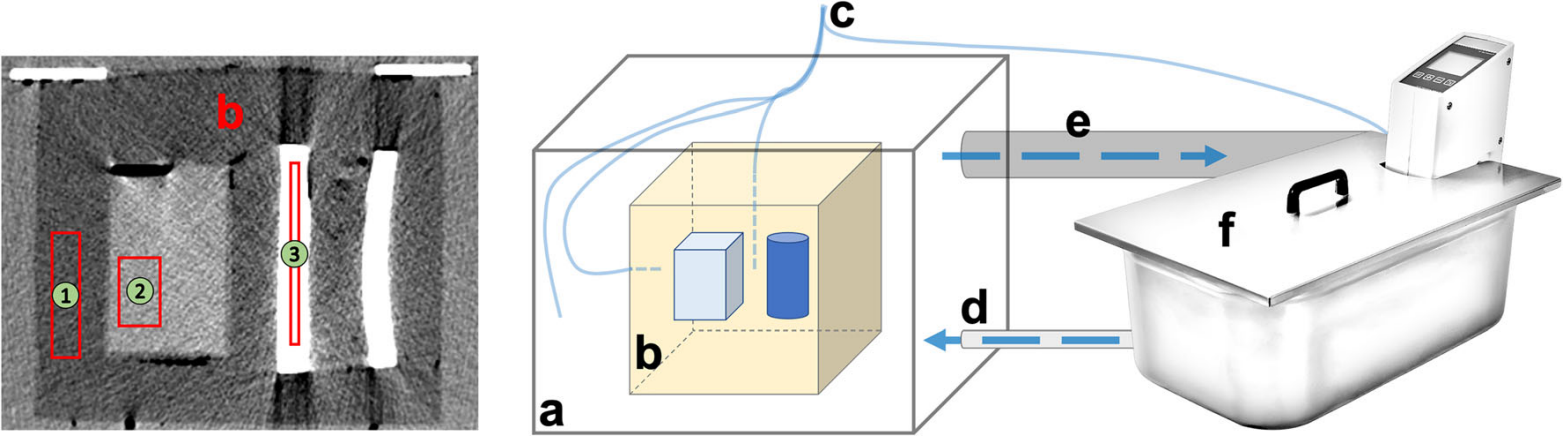
*Left* virtual monoenergetic image (VMI) at 70 keV of the tissue-equivalent phantom with rectangle regions of interest (ROIs) for measurement of fat-equivalent (beeswax, 1), muscle-equivalent (salty water solution, 2) and bovine bone (3). *Right* temperature-regulating water circuit system with (a) phantom housing container with fixture for the (b) tissue-equivalent phantom and (c) temperature probes. Water is pumped into the phantom housing container through (d) a silicone hose, (e) an overflow pipe returning the water back into the (f) water bath with heater (0–100 °C) and pump. During a series of measurements, the phantom container and the water bath were covered by lids so that the system constituted a closed circuit.

A temperature-regulating water circuit system (Fig. 2) was developed for the systematic evaluation of the correlation of temperature and CT number. More than 35 L of water was heated in a water bath (Thermoboy c20, mgw Lauda) and subsequently pumped through a silicone hose into the phantom housing container. The phantom container features an overflow port located above the top edge of the phantom. Consequently, it is guaranteed that the phantom is completely immersed by water. When it reaches the overflow port, the water flows back into the water bath basin. This measurement setup constitutes a closed system; thus, the temperature can be kept constant over a long period of time. The temperature of the system was monitored by four T1 fiber-optic temperature probes (Rugged Fiber Optic Temperature Monitoring System). In the top center of the phantom is a 7.50-cm-deep blind hole that accommodates a temperature probe (probe 1) for monitoring the phantom’s core temperature. Another temperature probe (probe 2) is located on the side at a depth of 2 cm in the phantom. To avoid the risk of water infiltrating and thus distorting the temperature measurement, both holes were sealed with beeswax. Two additional temperature probes were located directly in the water in the phantom housing tank (probe 3) and in the water bath (probe 4).

### CT measurements and evaluation

The measurements were performed in a temperature range from 18 up to 50°C (step size approx. 5°C) on a Revolution CT (GE Healthcare) by means of the GSI technology (voltage 80 kV and 140 kV, current 235–360 mA SmartmA, helical scan type, pitch 0.992). Complementary measurements were performed without GSI (voltage 120 kV, current 130–545 mA, helical scan type, pitch 0.992). Measurements were repeated (*n* = 14) for each temperature and CT protocol. To increase the reliability, the measurements were performed in thermodynamic equilibrium of the phantom. This required a waiting time of 4 to 10 h between measurements.

The raw data were reconstructed by FBP (= ASIR-V 0%), ASIR-V (50%, 100%) and DLIR-L (lowest filtering), DLIR-M (medium filtering) and DLIR-H (highest filtering) with the standard kernel and an image matrix of 512 × 512 pixels. For the evaluation of the GSI raw data, VMI at 40 keV, 50 keV, 60 keV, 70 keV, 80 keV, 100 keV, 120 keV and 140 keV were used. Additionally, the kernels bone, boneplus, soft and detail were examined for polychromatic images and GSI (VMI at 40 keV, 70 keV and 120 keV) with ASIR-V 100%. The standard kernel had to be used for DLIR.

For each measurement, the average HU of rectangle regions of interest (ROIs) with mean areas of 8.24 cm^2^ for fat (25 × 96 pixels) and muscle (40 × 60 pixels) and 2.58 cm^2^ for bone (5 × 150 pixels) was analyzed (Fig. 2 left). The ROIs were set automatically; thus, the position of the ROIs was exactly at the same position for all reconstructions. The dependency of the average CT numbers as a function of temperature was analyzed using linear regression analysis. The software SPSS Statistics, version 26 (IBM), was used for statistical evaluation. The 95% and 50% confidence intervals (CI) were calculated for the temperature sensitivity.

## Results

**Fig. 3:**
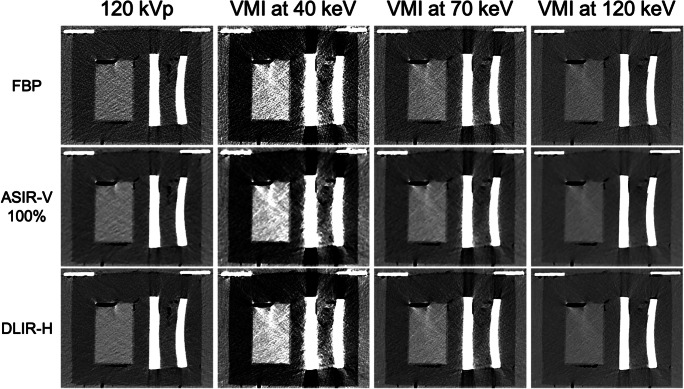
Polychromatic 120-kVp images and virtual monoenergetic images (VMI) with three types of reconstruction (FBP, ASIR-V 100%, DLIR-H) are shown.

The measurement setup enabled reproducible measurements. During the measuring process, no movement artefacts caused by the circulating water system were observed (Fig. 3). The bone, in parts, showed beam hardening artefacts. The measurement uncertainty due to temperature gradients between the margin of the phantom and the core center was 0.59 ± 0.37 °C (temperature probes 1 and 2). The difference between water temperatures in the water bath and phantom housing container was 0.37 ± 0.28 °C (temperature probes 3 and 4).

**Fig. 4:**
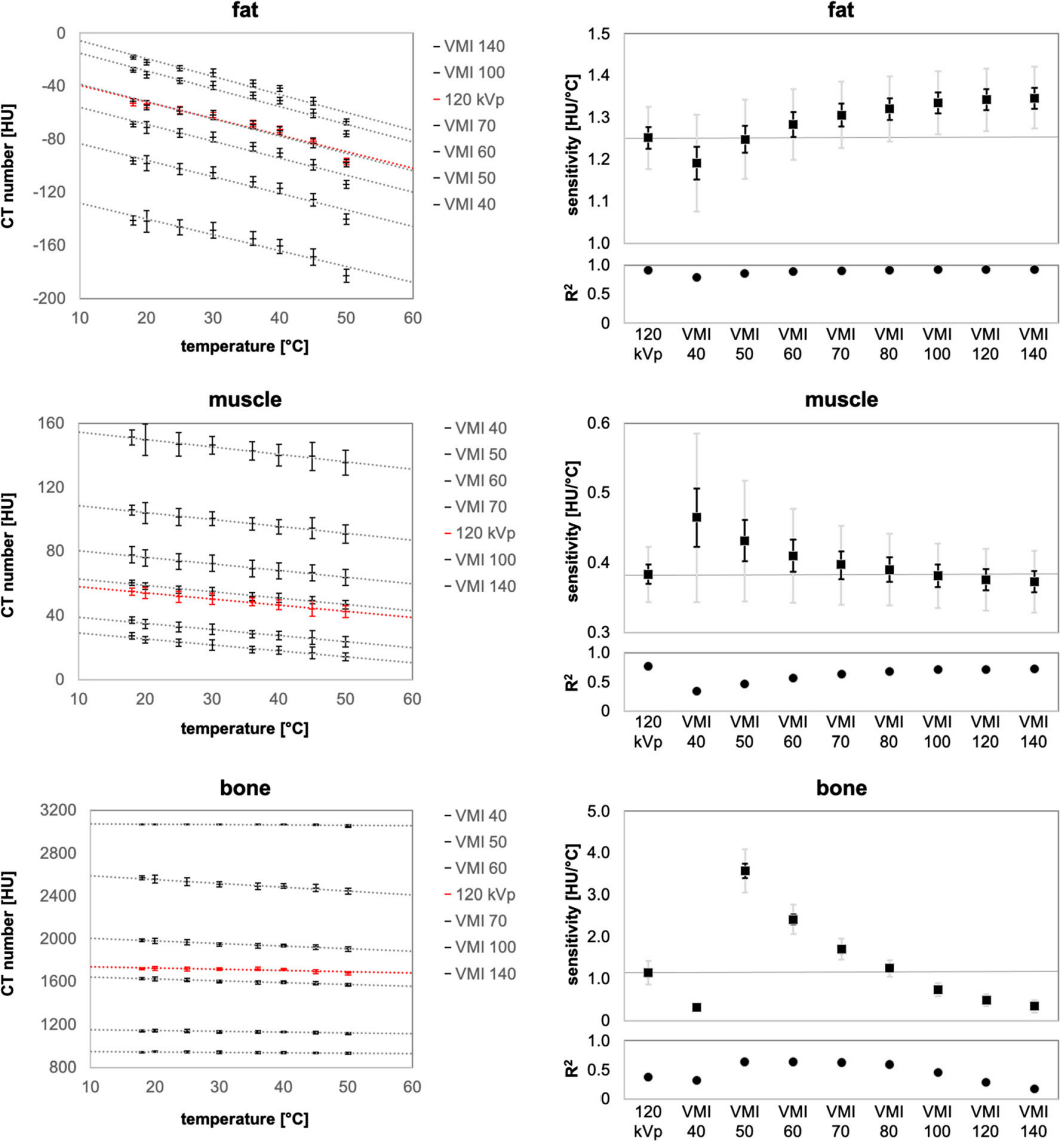
*Left* dependency of the CT number on the temperature with linear regression analysis for virtual monoenergetic images (VMI) and polychromatic 120-kVp images. *Right* results of temperature sensitivity with 95% (gray) and 50% (black) confidence interval (CI) and coefficient of determination for fat, muscle and bone.

The regression analysis showed a significant inverse linear dependency between temperature and average CT number (Fig. 4 left). Depending on the monoenergy, the VMI show a higher temperature sensitivity compared to polychromatic images (Fig. 4 right). For fat, the temperature sensitivities were 1.25 HU/°C (95% CI 1.18–1.33) for 120 kVp, 1.19 HU/°C (95% CI 1.08–1.31) for VMI at 40 keV and 1.35 HU/°C (95% CI 1.27–1.42) for VMI at 140 keV. For muscle, the temperature sensitivities were 0.38 HU/°C (95% CI 0.34–0.42) for 120 kVp, 0.47 HU/°C (95% CI 0.34–0.59) for VMI at 40 keV and 0.37 HU/°C (95% CI 0.33–0.42) for VMI at 140 keV. For bone, the temperature sensitivities were 1.15 HU/°C (95% CI 0.87–1.43) for 120 kVp, 3.58 HU/°C (95% CI 3.07–4.09) for VMI at 50 keV and 0.35 HU/°C (95% CI 0.20–0.49) for VMI at 140 keV.

**Fig. 5:**
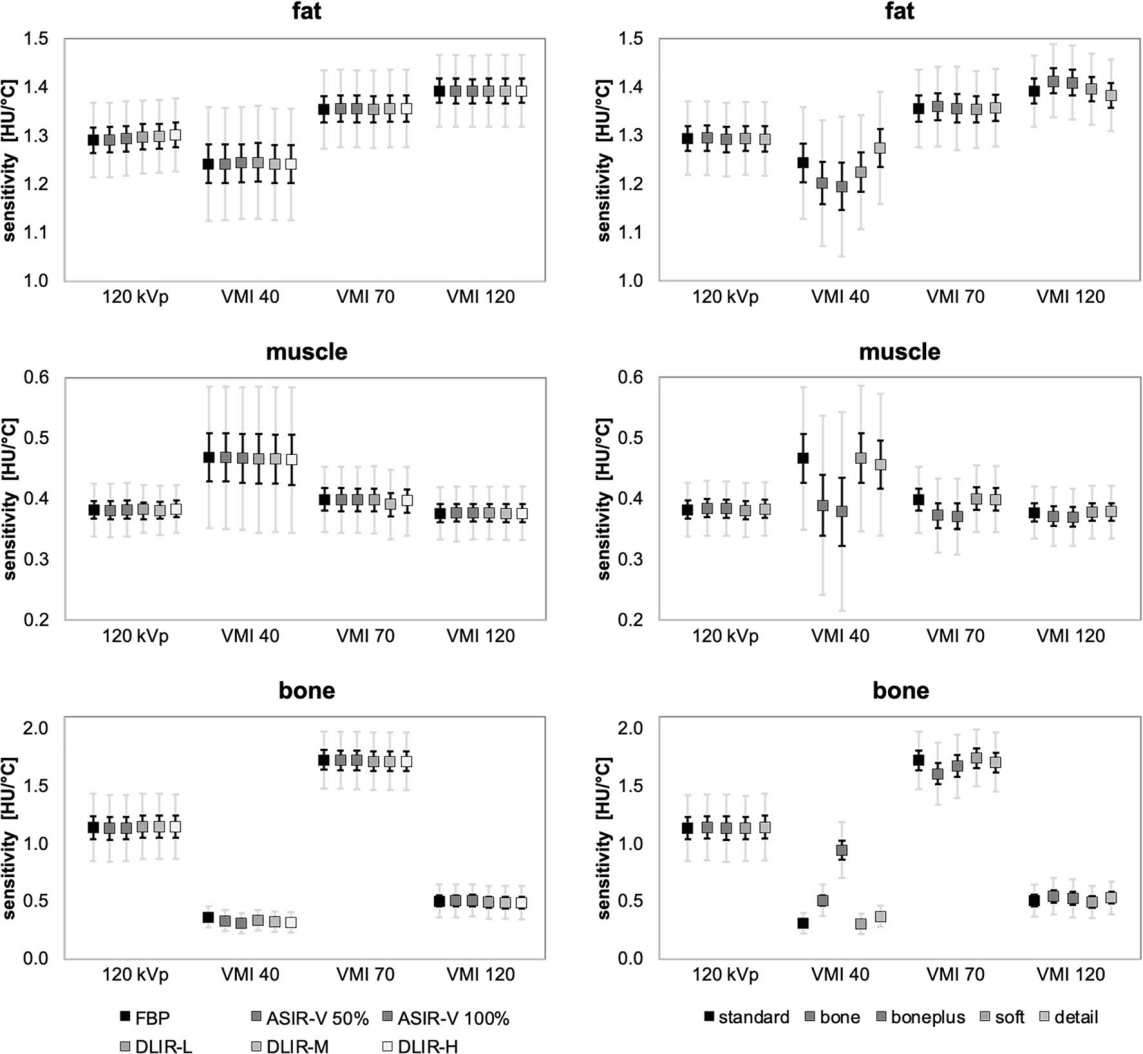
Results of temperature sensitivity with 95% (gray) and 50% (black) confidence interval (CI) for *left* different reconstruction types (FBP, ASIR-V 50%, ASIR-V 100%, DLIR-L, DLIR-M, DLIR-H) and *right* different reconstruction kernels (standard, bone, boneplus, soft, detail).

The application of ASIR-V and DLIR had no significant impact on the temperature sensitivity (Fig. 5 left). The reconstruction kernel has no effect on conventional polychromatic 120-kVp measurements (max difference 0.01 HU/°C); however, it can affect the temperature sensitivity for VMI (Fig. 5 right). For fat, the temperature sensitivity varies between 0.01 HU/°C (VMI at 70 keV) and 0.08 HU/°C (VMI at 40 keV: standard 1.19 HU/°C, bone 1.15 HU/°C, boneplus 1.15 HU/°C, soft 1.17 HU/°C, detail 1.23 HU/°C; VMI at 120 keV: standard 1.34 HU/°C, bone 1.36 HU/°C, boneplus 1.36 HU/°C, soft 1.35 HU/°C, detail 1.33 HU/°C). For muscle, compared to the standard kernel, only the bone kernel has a negative influence on the temperature sensitivity (VMI at 40 keV: standard 0.47 HU/°C, bone 0.39 HU/°C, boneplus 0.38 HU/°C; VMI at 70 keV: standard 0.40 HU/°C, bone 0.37 HU/°C, boneplus 0.37 HU/°C; VMI at 120 keV: standard 0.38 HU/°C, bone 0.37 HU/°C, boneplus 0.37 HU/°C). In contrast, the bone kernel may have advantages for representation of bones (VMI at 40 keV: standard 0.31 HU/°C, bone 0.51 HU/°C, boneplus 0.94 HU/°C, soft 0.30 HU/°C, detail 0.37 HU/°C; VMI at 70 keV: standard 1.72 HU/°C, bone 1.60 HU/°C, boneplus 1.67 HU/°C, soft 1.74 HU/°C, detail 1.70 HU/°C; VMI at 120 keV: standard 0.51 HU/°C, bone 0.55 HU/°C, boneplus 0.53 HU/°C, soft 0.50 HU/°C, detail 0.53 HU/°C).

## Discussion

For evaluating the sensitivity of main human tissues in CT-based thermometry, our study is one of the first integrating VMI. VMI proved suitable for CT-based thermometry and enable a higher temperature sensitivity (fat 8%, muscle 24%, bone 211%) compared to conventional polychromatic 120-kVp measurements. In addition, with conventional polychromatic measurements, there are no parameters to influence the temperature sensitivity. Tan et al [12] in 2019 did not find any statistically significant difference in CT numbers with varied detection parameters for the same temperature setting, so that the scan parameters such as, e.g., voltage, tube current, pitch, gantry rotation time and slice thickness had no influence on the result of thermometry. With VMI, we found new parameters to modulate the temperature sensitivity. An interesting point is that, depending on the type of tissue, a certain monoenergy should be preferred (fat VMI at 140 keV, muscle VMI at 40 keV and bone VMI at 50 keV). Furthermore, additional reconstructions can be obtained by dual-energy measurements. This leads to more detailed information about the material composition, e.g. material density images and data files (see Fig. 1 options). However, VMI partly shows larger fluctuation of CT numbers, due to a slightly larger CI for the temperature sensitivity (Fig. 4 right). One reason could be beam hardening artefacts of bone, which also affect the measurements of fat and muscle. Separate calibration curves, depending on the tissue and monoenergy of the VMI, are required.

For clinical use, CT-based thermometry benefits from modern reconstruction algorithms such as ASIR-V and DLIR, which can drastically reduce the radiation dose [14, 16]. These kinds of reconstruction algorithms and the modification of other protocol parameters have no negative influence on CT numbers [12, 14, 17]. However, ASIR-V and DLIR reconstruction methods can reduce beam hardening artefacts (e.g. of bones) and thus improve the result for CT-based thermometry. Furthermore, the image noise can be drastically reduced, allowing more stable CT numbers for smaller ROIs.

The DLIR setup currently just works with the standard kernel. In future, more kernels will be added for DLIR from manufacturer, which should provide clear statements to kernel influences on CT-based thermometry in further investigations. A major problem of CT-based thermometry is the reproducibility of the results [13]. For this reason, we obtained multiple measurements for our investigations. The numerous repetitions lead to very reliable and stable correlations between temperature and CT number. In clinical application of CT-based thermometry, a slice should be measured multiple times to average the CT numbers.

The measured temperature sensitivity for the muscle-imitating salt-water solution corresponds to literature sources [4, 5]. Fat density varies naturally between 0.90 and 0.97 g/cm^3^ [23]. Therefore, the homogeneous beeswax (density 0.95 g/cm^3^) is used as a homogeneous tissue equivalent to avoid influences due to density changes. We measure CT numbers between −53 HU and −97 HU for a temperature range from 18 to 50 °C. Porcine fat tissue samples, as comparable biotic tissue, show CT numbers between −105 and −135 HU for a temperature range from 20 to 45 °C [24]. The measurements with beeswax are limited to 50°C. Hot temperatures lead to rapid melting and faster changes in CT numbers than predicted by the linear regression (Fig. 4 left). Excluding the critical 50°C measurement, the temperature sensitivity is 1.02 HU/°C (120 kVp) and 1.13 HU/°C (VMI at 140 keV) for fat. Thus, VMI perform 11% better compared to 120 kVp.

There are some limitations to the present study. One of the major limitations is that the experiments were performed on a tissue-equivalent phantom. For example, physiological processes (e.g. blood perfusion) may cause local alterations in data points due to thermal conductivity and heat redistribution. In addition, we could not heat the phantom above 50 °C, due to the melting point of beeswax. However, it is possible to use the linear regression shown in Fig. 4 to estimate the CT numbers at higher temperatures.

In conclusion, with the application of dual-energy and VMI, CT-based thermometry has parameters to influence and improve temperature sensitivity. Using ASIR-V and DLIR has no negative influence on the results, opening up the potential of drastic dose reductions. The temperature-regulating water circuit system enables reproducible measurements of CT numbers as a function of temperature, e.g. for bone, fat and muscle tissue equivalents. The developed setup is a groundwork for further research concerning complex test objects regarding CT-based thermometry.

## Abbreviations

ASIR-V: Adaptive statistical iterative reconstruction-Volume
CI: Confidence interval
DLIR: Deep learning image reconstruction
FBP: Filtered back projection
GSI: Gemstone spectral imaging
VMI: Virtual monoenergetic images

## Declarations

### Guarantor

The scientific guarantor of this publication is Andreas Heinrich.

### Statistics and Biometry

At least one of the authors (Andreas Heinrich) has significant statistical expertise.

### Methodology

• prospective

• experimental

• performed at one institution
